# Exploring the transcriptome of *Staphylococcus aureus* in its natural niche

**DOI:** 10.1038/srep33174

**Published:** 2016-09-19

**Authors:** Diego Chaves-Moreno, Melissa L. Wos-Oxley, Ruy Jáuregui, Eva Medina, Andrew PA Oxley, Dietmar H. Pieper

**Affiliations:** 1 Microbial Interactions and Processes Research Group, Helmholtz Centre for Infection Research, Inhoffenstr. 7, 38124 Braunschweig, Germany; 2 Infection and Immunology Research Group, Helmholtz Centre for Infection Research, Inhoffenstr. 7, 38124 Braunschweig, Germany

## Abstract

*Staphylococcus aureus* is an important human pathogen and commensal, where the human nose is the predominant reservoir. To better understand its behavior in this environmental niche, RNA was extracted from the anterior nares of three documented *S. aureus* carriers and the metatranscriptome analyzed by RNAseq. In addition, the *in vivo* transcriptomes were compared to previously published transcriptomes of two *in vitro* grown *S. aureus* strains. None of the *in vitro* conditions, even growth in medium resembling the anterior nares environment, mimicked *in vivo* conditions. Survival in the nose was strongly controlled by the limitation of iron and evident by the expression of iron acquisition systems. *S. aureus* populations in different individuals clearly experience different environmental stresses, which they attempt to overcome by the expression of compatible solute biosynthetic pathways, changes in their cell wall composition and synthesis of general stress proteins. Moreover, the expression of adhesins was also important for colonization of the anterior nares. However, different *S. aureus* strains also showed different *in vivo* behavior. The assessment of general *in vivo* expression patterns and commonalities between different *S. aureus* strains will in the future result in new knowledge based strategies for controlling colonization.

*Staphylococcus aureus* is recognized as a major human pathogen, but also described as a human commensal. The human nose is its major reservoir and ecological niche where approximately 20–30% of humans are reported to be permanent carriers^1^. Even though carriage is usually asymptomatic, nasal carriage has a crucial function as a source of invasive infections in both community and hospital settings^2^. In recent decades, the prevalence of methicillin-resistant *S. aureus* (MRSA) has increased markedly^3^,^4^ and vancomycin resistant clones have also been reported^5^. The reasons for the rapid emergence of MRSA are still unclear and the spread cannot be explained solely be the antibiotic selection pressure^6^. In how far the natural ecological niche constitutes a selective advantage for MRSA remains to be elucidated. *S. aureus* carriage is influenced by host and environmental factors^7^ and also by interactions with other community members within the anterior nares^8^,^9^. Thus, it is assumed that a better understanding of the ecology of this niche may support strategies for limiting the carriage of *S. aureus*^8^,^10^,^11^.

*S. aureus* clearly belongs to the most studied bacterial species and its behavior under different *in vitro* conditions has been analyzed in great detail, where virulence factors^12^,^13^, global regulation^14^,^15^, stress response^16^, nutrient acquisition^17^, immune evasion^18^ and attachment mechanisms^12^, among others have been described. However, with the emergence of high throughput sequencing methods, the interest to understand the *in vivo* situation and the interactions between the host and the inhabiting bacterial communities has increased significantly^19^,^20^ and recent studies using deep sequencing transcriptomic analysis (RNAseq) indicate the behavior of *S. aureus in vivo* to be significantly different from that observed by the classical *in vitro* approach^20^,^21^.

Efforts to understand the survival and persistence of *S. aureus* in its ecological niche on the one hand aimed to understand the metabolic challenges. The analysis of nasal secretions resulted in the development of a synthetic nasal medium, which should mimic the nasal environment^22^. Thereby, iron limiting conditions as well as the absence of distinct amino acids were identified as shaping the transcriptional response. However, even though the use of such a medium may offer important insights into the global reaction of *S. aureus* to its natural conditions, it evidently cannot simulate the host environment and simulate e.g. adhesion processes^19^,^23^. Moreover the anterior nares are a complex ecosystem, which is characterized not only by the interaction of *S. aureus* with the host, but by the presence of a complex microbial community^8^,^10^ which may compete for nasal nutrients and interact with *S. aureus.* All these interactions cannot be easily mimicked *in vitro*.

In the current report, we analyzed the genome-wide transcriptional activity of *S. aureus in vivo* in the anterior nares of 3 volunteers at two different time points by RNAseq and compared the transcriptome profiles to those previously obtained by two distinct *S. aureus* strains under *in vitro* conditions^24^. The results of our study provide the first high-resolution analysis of the transcriptional response of *S. aureus* to its natural environment and will allow an understanding of how *S. aureus* persists in such a hostile environment niche.

## Results and Discussion

### The bacterial community of three *S. aureus* carriers

To gain insights into the behavior of *S. aureus* in its natural ecological niche, three healthy volunteers persistently colonized by *S. aureus* were selected. Volunteer 1 was colonized by a novel strain (novel spa type with repeat succession 15-21-12, *S. aureus* D1), volunteer 2 by a strain typed as spa type t0254 (*S. aureus* O1) and volunteer 3 by a strain typed as spa type t12 (*S. aureus* R1). Spa type t12 is reported to be one of the most abundant types colonizing healthy individuals^25^, specifically in young adults as is the case here. Multilocus sequence typing identified *S. aureus* D1 and R1 to belong to sequence type ST30 and *S. aureus* O1 to belong to sequence type ST15.

**Figure 1:**
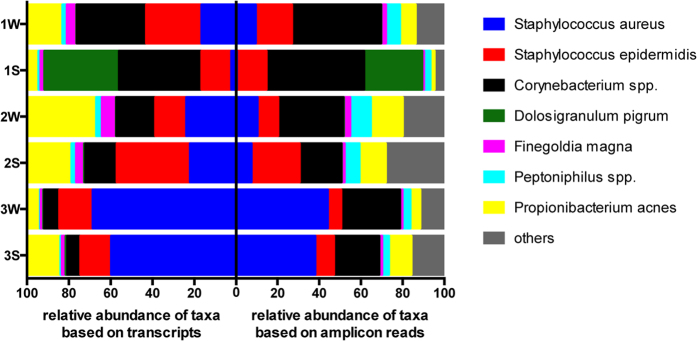
Description of the microbial community of three volunteers at two different time points during the year. The graphic show the relative abunance per taxonomic group. The right side shows the abundance of species based on the sequencing of 16S rRNA amplificons (V1-V2), whereas the left side shows the abundance based on the amount of metatranscriptomic reads assigned to this species.

The bacterial communities of the anterior nares of these carriers were sampled during both winter (W) and summer (S) and the composition of the active bacterial community analyzed by deep sequencing of 16S rRNA amplicons following reverse transcription–polymerase chain reaction (RT–PCR) of total RNA extracts (Fig. 1, right). Analysis revealed a high abundance of *S. aureus* sequence reads in 5 of the 6 samples, and only in volunteer 1 it comprised <1% of sequence reads during summer (Volunteer 1S). A specifically high amount of sequence reads originating from *S. aureus* was observed in volunteer 3 (38–44%) indicating this organism to be specifically active in this person.

Overall, all three volunteers showed the presence of a bacterial community composed of core nasal colonizers such as *Corynebacterium accolens/tuberculostearicum* (19–42% relative abundance), *Staphylococcus epidermidis/capitis/caprae* (6–23%), *Propionibacterium acnes* (2–15%) or *Peptoniphilus* sp. (3–9%) among others (Fig. 1 right and Supplementary Dataset S1). While the composition of the bacterial communities of volunteer 2 and 3 were very similar during both sampling times (Fig. 1 right), evident differences were observed for volunteer 1, where the winter sampling showed a mild colonization by *S. aureus* (9%), whereas the summer community was dominated by *Dolosigranulum pigrum* and *Corynebacterium propinquum/ pseudodiphtheriticum.*

### Metatranscriptomic analysis of nasal microbial communities

The same six *in vivo* samples described above were subject to a metatranscriptomic analysis by RNAseq. After quality filtering, 9–106 million sequence reads per sample were obtained (Table 1). Between 15 and 96% of those reads could be assigned to human RNA and were removed. After removal of 16S rRNA reads between 2 and 8 million of sequence reads remained as potential bacterial mRNA (Table 1).

A crucial step for evaluating the activity of *S. aureus* in its natural niche is the capability to separate sequence reads originating from *S. aureus* and *S. epidermidis*. Using a database of 25 *S. aureus* and 2 *S. epidermidis* genomes to assign reads to these species, between 12,000 and 120,000 reads showed a high similarity of (alignment length) * (% identity/ query length) ≥80% with *S. aureus* genomic sequences. However, of those reads between 35 and 95% could also be assigned to *S. epidermidis,* showing the similarity between the two species and the high level of possible mis-assignment. In contrast, using a similarity ratio ≥90%, the percentage of reads that could not be clearly assigned to one of those species decreased substantially to 9–34%. Only in case of sample 1S, 90% of the reads remained of unclear assignment (Table 1). This corresponds to the high relative abundance of *S. epidermidis* compared to that of *S. aureus* in this specific sample, as indicated by amplicon sequencing (Fig. 1 right). Thus, assignment of reads to *S. aureus* and *S. epidermidis* was performed using a similarity ratio ≥90% as cut-off, whereas assignment to bacterial genera was performed with a ≥80% similarity ratio as cut-off (Table 2).

In total, between 18,000 and 310,000 reads could be assigned to the different bacterial genera and species, which is only a subset of the metatranscriptomic reads remaining after depletion of human and ribsosomal reads (Table 2). The remaining unassigned mRNA reads illustrate the complexity of the anterior nare niche, and may represent transcripts pertaining to other community species such as eukaryotic species like fungi and protozoa^26^, although identifying these taxa was beyond the scope of this work. However, previous experiences also have shown an association between the sample quality and the number of RNAseq mapped reads obtained from it, *i.e.* samples with lower quality, and therefore more degraded RNA molecules, as present in *in vivo* samples yield both lower numbers of reads that can be assigned and reads mapped to genes^27^ and may explain why only a subset of metatranscriptomic reads could in fact be assigned here.

Comparison of the composition across the six nasal microbial communities under study as deduced by amplicon sequencing (Fig. 1 right) versus the composition as indicated by the relative amounts of transcripts assigned to the different species and genera (Fig. 1 left) indicated similar structures. This in turn indicates that representative amounts of metatranscriptomic reads were sampled in each case.

Potential *S. aureus* reads were then mapped against a database of orthologous groups of proteins previously built on the basis of 25 reference genomes^24^ and 73–80% of the reads could be successfully mapped to the *S. aureus* gene catalogue (Table 1). The remaining reads matched intergenic regions that mainly reflect the sites of transcription initiation and different RNA species. Analysis of the sequence reads that could be mapped to *S. aureus* versus those that could be mapped to both *S. aureus* or *S. epidermidis* considering a ≥90% similarity ratio showed, that for some genes all reads fall into the last category, indicating the presence of highly conserved gene regions, preventing a clear assignment (see Supplementary Dataset S2). As sample 1S mainly contains *Staphylococcus* sequence reads, which could not clearly be assigned, it was excluded from further detailed analysis keeping the samples in which *S. aureus* was shown to be active and abundant.

### Functional categorization of transcripts expressed by *S. aureus* under *in vivo* conditions

**Figure 2:**
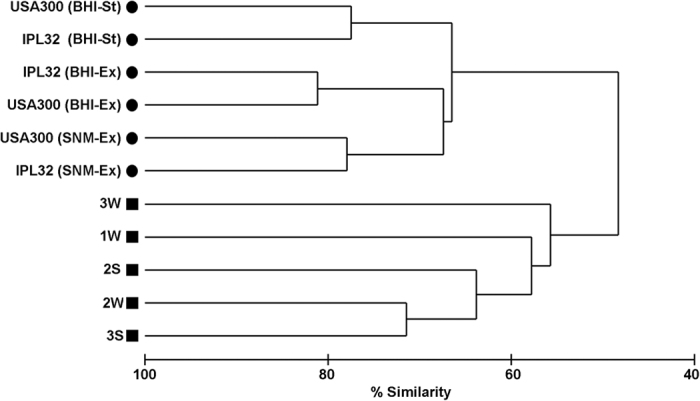
Cluster analysis within *in vitro* and *in vivo* conditions. Dendrogram constructed by agglomerative hierachical clustering (group-average) based on a relative abundance matrix constructed from comparisons of metatransciptomic data between five *in vivo* samples analyzed here (depicted by filled squares) and *in vitro* transcriptomic data generated previously^24^ using *S. aureus* USA300 LAC and *S. aureus* IPL32 growing in BHI or SNM at different growth phases (exponential - Ex and stationary - St). The percentage similarity between conditions was calculated using the Bray-Curtis similarity algorithm.

Due to the difference in sequencing depth between the samples, random resampling was performed and the expression patterns analyzed on the basis of 5,000 sequence reads each (Supplementary Dataset S2 and Supplementary Fig. S1). These *in vivo* expression patterns were compared to those previously reported for *S. aureus* USA300 LAC and *S. aureus* IPL32 grown *in vitro* in rich medium (BHI) and a synthetic nasal medium (SNM)^24^ which mimics the nasal conditions^22^. Cluster analysis was used to determine the similarity between the global transcriptional profiles of *S. aureus* under the different conditions. The dendrogram (Fig. 2) shows that all 5 *in vivo* transcriptomes differed substantially from those of USA300 LAC and *S. aureus* IPL32 under *in vitro* growth conditions, that is, these two groups of samples were <50% similar, and those of both strains grown in synthetic nasal medium (SNM) were not more similar to the *in vivo* trancriptomes then those obtained in rich medium.

It is important to note that the *in vitro* RNAseq libraries had been amplified using Ambion’s MessageAmp kit^24^, while the *in vivo* libraries in this work were generated using the Epicentre’s ScriptSeq Kit, but where isolation of RNA and sequencing were performed in the exact same manner. A comparison between these two methods on *S. aureus* cells grown to mid-log phase revealed only an additional 10% difference in the global expression profile compared to replicates (taking into account standardized read count across 2,582 *S. aureus* genes), where the rank-order of gene expression was mostly retained (Spearman rank correlation of 0.911) (data not shown).

Immense differences were obvious between the 5 *in vivo* transcriptomes sharing only >55% of similarity. Differences were observed not only between *S. aureus* transcriptomes from different individuals but also between transcriptomes obtained from the same volunteer at different timepoints, and thus from the same *S. aureus* strain. This reveals that differences in the transcriptomic profiles are not only due to the colonizing strain, but also to the environment provided by the host.

Gene expression analysis based on the relative abundances of gene transcripts assigned to their respective Clusters of Orthologous Groups (COG) showed no evident difference between *in vivo* and *in vitro* conditions (Supplementary Fig. S2), and mainly differences in the general distribution per categories between growth media were obvious, as previously reported^24^. However, differences were also visible in the relative transcript abundances of genes involved in inorganic ion transport and metabolism (category P) and secondary metabolites biosynthesis, transport and catabolism (category Q), where levels during growth in complex medium were lower compared to those during growth in either SNM or *in vivo*.

To obtain a better view on differences in transcriptomes, genes that were expressed to levels exceeding a normalized value of 1000 rpm after resampling were considered in more detail (Supplementary Dataset S2). Of the genes that were expressed in at least one *in vivo* sample at a level exceeding 1000 rpm, 240 genes were indicated to be expressed differently under *in vivo* versus *in vitro* conditions (see Materials and Methods and Supplementary Dataset S3).

### Expression of genes encoding surface bound proteins

**Figure 3:**
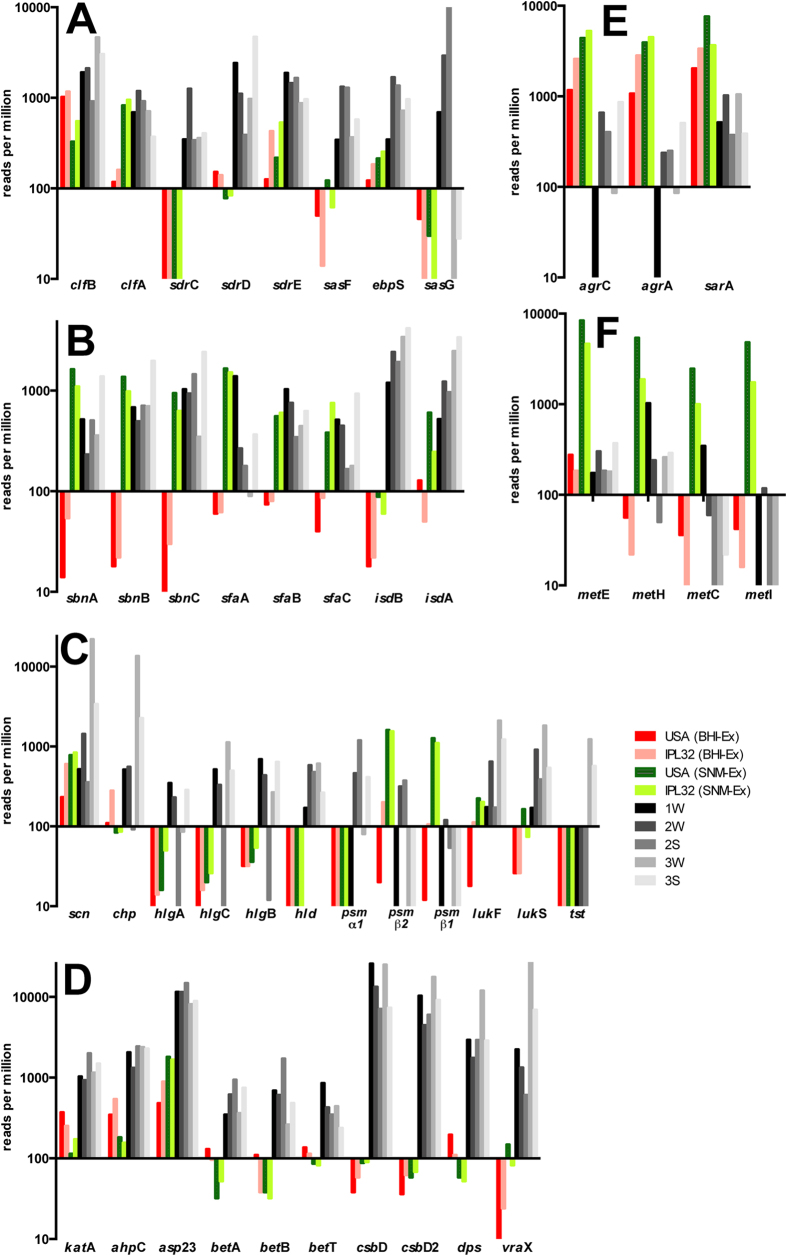
Comparison between the expression of various *S. aureus* survival factors. Expression of genes encoding different adhesion factors (**A**), of genes involved in iron homeostasis (**B**), of genes involved in subversion of the host defense (**C**), of stress response genes (**D**), of genes encoding regulators (**E**) and methionine biosynthesis genes (**F**) of colonizing *S. aureus* strains *in vivo* (grey scaled) compared to their expression by *S. aureus* USA300 LAC or IPL32 during exponential growth *in vitro* on BHI or SNM as previously decribed^24^. Expression levels are given as rpm (reads per million) of total reads.

The adherence to components of the human extracellular matrix is a key step for the persistence of *S. aureus* in the nasal habitat and a total of 35 adhesins have been examined in detail^28^. Of these, the staphylococcal cell-wall protein clumping factor B (ClfB, USA300HOU_2630) which promotes adhesion to squamous epithelial cells^29^ was indicated as important for nasal colonization^19^. In fact, read counts indicating transcription of the encoding gene *in vivo* varied from 910–4660 reads per million of total sequence reads (rpm), whereas those from cells grown in SNM (*in vitro*) were only 330–550 rpm (Fig. 3A). Reads assigned to *clf*A (USA300HOU_0819) were similar between *in vivo* and *in vitro* growth in SNM. However, *sdr*C, D and E, all characterized as encoding crucial attachment factors^13^, but yet to be linked with nasal colonization were all expressed to a much higher extent *in vivo* compared to their previously reported *in vitro* expression (e.g. 340–1260 rpm *in vivo* versus ≤10 rpm *in vitro* for *sdr*C) (Fig. 3A). This indicated the importance of SdrCDE (USA300HOU_0555-0557) as attachment factors for nasal colonization. Furthermore, the *sas*F gene (USA300HOU_2646) was expressed to a much higher extend *in vivo* compared to *in vitro* (340–1330 rpm versus 14–120 rpm, Fig. 3A).

The *Staphylococcus* surface proteins can interact with different compounds in the extracellular matrix, and also the elastin-binding protein (*ebp*S, USA300HOU_1419) was expressed to a higher extent *in vivo* compared to its *in vitro* expression (350–1690 rpm *in vivo* versus 210–250 rpm after growth in SNM), whereas the *cna* gene (SAA6008_02751) encoding collagen adhesin was expressed only in two of the three volunteers (samples of volunteer 1 winter, 1W; and of volunteer 3 in summer and winter, 3S and 3W). Most intriguingly was the behavior of *sas*G (SACOL2505), which has been reported to promote adhesion to nasal epithelial cells^13^,^30^,^31^. This gene was expressed *in vitro* by the two strains previously analyzed only at levels <50 rpm of reads, but reached roughly 10,000 rpm in volunteer 2S (Fig. 3A). As mentioned above, the majority of transcripts obtained from sample 1S could not clearly be assigned to *S. aureus* or *S. epidermidis*, however, of the 583 reads that were mapped exclusively to *S. aureus,* >50% were due to transcription of *sas*G. Taken collectively, these levels of expression indicate that *S. aureus* uses a whole battery of adhesins to colonize the anterior nares, with the precise nature of the expression of adhesins being seemingly dependent on the colonizing strain and/or the human host.

### Expression of genes involved in iron homeostasis

Much of the success of *S. aureus* as a major colonizer across the human body has been attributed to its capacity to retrieve iron from the host^17^,^32^ and iron limiting conditions as evidenced by the high expression of iron-regulated genes have recently been observed in culture media mimicking the conditions in the anterior nares^22^,^24^ or human plasma among others^15^. In fact, both the genes encoding proteins for the biosynthesis of staphyloferrrin B (*sbn*ABCDEFGHI, USA300HOU_0127-0135) and staphyloferrin A (*sfa*CBAD, USA300HOU_2170-2173) as well as the respective transport systems (*sir*ABC, USA300HOU_0126-0124; *hts*ABC (USA300HOU_2169-2167). Similar to the previously described *in vitro* samples, expression of all four mentioned operons was high in all five *in vivo* samples indicating actual iron limitation governing the performance of *S. aureus in vivo* in the nares (see Fig. 3B and Supplementary Dataset S2).

High amounts of transcripts (up to 7060, 2740 and 5150 rpm, respectively in sample 3S) could be assigned to the *sit*ADB (USA300HOU_0651-0653) iron transporter, however, similar high expression of this transporter has previously been observed during stationary phase of growth^24^. Transcription of various other transport systems was observed (*fep*ABC, USA300HOU_0364-0366; *fhu*AB, USA300HOU_0668-0669; *sstA*BCD, USA300HOU_0759-0762), however, only in the case of the f*ep*ABC iron transport system, an increased expression *in vivo* compared to *in vitro* exponential growth in complex medium of strains USA300 LAC and ILP32 could be observed (see Supplementary Dataset S2).

Genes of the *isd*BACDEF gene cluster (SACOL1138, USA300HOU_1064-1068) encoding iron regulated surface determinants were also previously reported as being upregulated by *S. aureus* in SNM^22^,^24^,^32^. Even higher expression of these genes was observed here *in vivo* and as an example, *isd*B transcripts accounted for 1200–4160 rpm in *in vivo* transcriptomes and only 60–90 rpm after growth of strains USA300 Lac or IPL32 *in vitro* in SMN (Fig. 3B).

### Expression of genes required for subversion of the host defense

To survive within the host, *S. aureus* has developed various mechanisms to overcome the host immune defenses^33^. The staphylococcal complement inhibitor (SCIN), which interferes with all complement activation pathways is recognized as the most efficient complement inhibitor^18^ and the encoding *scn* gene has recently been shown to be highly expressed by *S. aureus* in an *in vivo* mouse model of infection^20^. During *in vitro* growth, its expression was typically relatively low and the level of transcripts never exceeded 1000 rpm^24^ (see also Fig. 3B) and low levels were also observed in a study comparing various *in vitro* conditions, where highest levels were observed during growth in human plasma^15^. Extreme differences were observed in the different volunteers and while in two volunteers expression levels were similar to those previously observed *in vitro*, in volunteer 3 transcript levels reached 22,000 rpm (Fig. 3B). Similarly the *chp* gene encoding chemotaxis inhibitory protein (USA300HOU_1947), where transcripts had been shown not to exceed 300 rpm *in vitro*^24^ was highly expressed in volunteer 3, reaching 13,600 rpm in sample 3W.

*S. aureus* can also secrete various exotoxins that damage the host cell plasma membrane, among them α-hemolysin, β-hemolysin, the bi-component leukocidins and γ-hemolysin and the phenol soluble modulins as well as δ-hemolysin^34^. While α- and β-hemolysin encoding genes (*hly*, USA300HOU_1099; *hlb*, SACOL2003) were expressed *in vivo* to a similar extent to previously described *in vitro* conditions, all of the *in vivo* samples (with the exception of 2S) showed a higher expression of *γ*-hemolysin encoding genes (*hlg*ABC, USA300HOU_2402, 2405, 2404) and two of the volunteers (volunteer 2 and 3) showed expression of the δ-hemolysin encoding gene (*hld*, USA300HOU_2031), expression of which was not observed *in vitro* (Fig. 3C)^24^. δ-Hemolysin is encoded within RNAIII^35^ which is part of the *agr* regulon and belongs to a group of small peptide toxins known to activate, attract and lyse neutrophils, which comprise also the phenol soluble modulins (PSMs). Both volunteers 2 and 3 in addition to *hld* also expressed the gene encoding cytosolic toxin PSMα1 (see Fig. 3C). However, in contrast to the expression of PSMβ1 and PSMβ2 under *in vitro* conditions in nasal medium^22^ with amounts of 1550–1610 rpm and 1100–1270 rpm, respectively by *S. aureus* IPL32 and *S. aureus* USA300 LAC^24^, their expression *in vivo* was only observed in one volunteer at levels not exceeding 370 and 120 rpm, respectively (Fig. 3C). This observation is in accordance with previous analysis, where expression of PSMβ was observed only *in vitro*^20^. Among the bi-component toxins not only were *hlg*ABC shown to be expressed *in vivo*, but high expression of a gene encoding a putative leucocydin (lukFS, USA300HOU_2011/USA300HOU_2013) specifically in volunteer 3 was observed, the importance of which remains to be elucidated.

Superantigens are virulent polypeptides that are capable of causing nonspecific T cell activation by circumventing normal antigen processing in the human host. The *tst* gene encoding toxic shock syndrome toxin-1 (TSST-1) has recently been observed in 15% of nasal *S. aureus* isolates from medical students in Poland^36^. Interestingly, the *tst* gene (SAV2011) was not only present in at least one volunteer, it was also expressed in this volunteer (volunteer 3) at levels reaching 1230 rpm (Fig. 3C).

### Expression of genes involved in stress response

Reactive oxygen species are produced by bacteria as a by-product of aerobic growth but also by the host, specifically as part of an oxidative killing mechanism^37^. Protection of *S. aureus* from such oxidative stress is mediated by a battery of enzymes among them KatA catalase and AhpC alkylhydroperoxide reductase, which both have been described as essential for nasal colonization by scavenge exogenously or endogenously produced hydrogen peroxide^38^. Both *kat*A (USA300HOU_1277) and *ahp*C (USA300HOU_0404) were expressed at high levels *in vivo* (>930 and >1320 rpm, see Fig. 3D) of one order of magnitude higher compared to their levels after growth of USA300 LAC or IPL32 in SNM (*katA*<370 rpm, a*hpC* <540 rpm), indicating *S. aureus* to experience oxidative stress in the anterior nares. Another gene highly expressed *in vivo* was *asp*23 (USA300HOU_2175, Fig. 3C). Asp 23 was initially described as a protein that accumulates after alkaline shock^39^ and has recently been identified as a membrane associated protein^16^ which may be important for cell envelope homoeostasis, specifically under adverse environmental conditions. In accordance with the previously observed high abundance of this protein in the complete proteome of *S. aureus*^16^, a high expression (480–1800 rpm), had been observed *in vitro*^24^, however, even when taking into account that 30–49% of reads could also be mapped to *S. epidermidis* (see Supplementary Dataset S2), higher expression levels were observed *in vivo* indicating Asp23 to be of major importance for survival of *S. aureus* in the host.

*S. aureus* has been shown to accumulate the compatible solute glycine betaine in response to osmotic stress^40^. A common bioysynthetic pathway for glycine betaine is its formation from choline, which in *S. aureus* is catalyzed by a choline dehydrogenase BetA forming glycine betaine aldehyde and a glycine betaine aldehyde dehydrogenase BetB forming glycine betaine^41^. Both *bet*A and *bet*B genes encoding the biosynthetic enzymes (USA300HOU_2605-2606) as well as *betT* encoding a choline transporter (USA300HOU_2610) were highly expressed *in vivo,* indicating *S. aureus* to experience osmotic stress in the nasal cavity (Fig. 3D).

CsbD is a bacterial general stress response protein, however, its role in stress response is unclear^42^. Typically, two *csb*D-like proteins are encoded in the genome of *S. aureus* strains, and both genes (USA300HOU_0868 and 1625) were expressed at levels under *in vivo* conditions two orders of magnitude higher (up to 17,640 and 25,860 rpm, respectively) compared to levels observed *in vitro* (<90 rpm) constituting two of the most abundant transcripts, indicating their importance for nasal colonization (Fig. 3D). Only 3–12% of the sequence reads attributed to *csbD2* expression in *S. aureus* could not clearly be assigned and may also originate from *S. epidermidis.* Similarly a *dps* family protein (USA300HOU_2128), a family comprising proteins protecting DNA under starved conditions, was expressed to a much higher extent *in vivo* compared to *in vivo* (up to 11,970 rpm).

Evidently, also genes encoding various membrane proteins (SAV0374, SAV0574, SAV1030 and SAV1359) were extremely differently expressed under *in vivo* versus *in vitro*^24^ conditions (Supplementary Dataset S2 and Fig. 3D). A further protein where the encoding gene showed a 2 order of magnitude difference in expression was VraX (Fig. 3D). *vra*X expression was also previously shown to be upregulated by multiple cell wall and/or membrane active compounds^43^,^44^,^45^ indicating that vraX up-regulation follows all forms of cell membrane and/or cell wall metabolism insult and that the anterior nares are a harsh environment for bacteria to survive.

### Expression of genes encoding regulatory systems

Regulation in *S. aureus* has been widely described *in vitro*^14^ and is performed by two-component regulatory systems (TCRS) and the SarA homologs, that commonly control many virulence factors^46^. Of the regulatory systems, substantial differences between *in vivo* and *in vitro* conditions were observed for the *agr*AC genes (USA300HOU_2035-2034) encoding the two-component AgrAC regulatory system as well as the *sar*A gene. While the *agr*AC genes represented 1070–4500 and 1170–5260 rpm, respectively, during growth of USA300 LAC and IPL32 strains *in vitro*^24^, they summed up to 510 and 860 rpm, respectively *in vivo* (Fig. 3E). Low expression of *agr* genes concomitantly with a high expression of virulence factors *in vivo* has been reported recently^20^ and some of the virulence factors assumed to be upregulated by the Agr or SarA system^47^, such as *γ*-hemolysin were in fact upregulated *in vivo* in the nose. Thus, these staphylococcal virulence factors may be under the control of additional regulatory mechanisms *in vivo*.

### Expression of genes involved in metabolic processes

The regulation of a wide variety of virulence factors its dependent on the nutrient availability^48^ and nasal secretions were analyzed previously with the objective to determine the abundance and limitations of potential nutrients that could be consumed by nasal communities, specifically *S. aureus*^22^. Methionine biosynthesis had been reported to be crucial for *S. aureus* to inhabit the nasal habitat, and genes encoding methionine biosynthetic enzymes such as cystathionine-*γ*-synthase (*met*I) and cystathionine-β-lyase (*met*C), as well as two L-methionine ABC-transport systems were reported to be up-regulated during growth in synthetic nasal medium^22^,^24^ and MetI was even reported to be indispensable for growth in SNM. However, while the USA300HOU_0376-USA300HOU_0380 *met*EHCI methionine biosynthesis operon was highly expressed in SNM, it was only poorly expressed during growth in rich medium as well as *in vivo* (Fig. 3F). In addition the strong upregulation of two L-methionine ABC-transport systems (*met*N1P1Q1, SACOL0504-0506 and *met*N2P2Q2 USA300HOU_0847 – 0849) previously observed in SNM^22^,^24^ did not occur *in vivo* (Fig. 3F), indicating the strong upregulation of methionine biosynthetic genes and the respective transport systems to be a medium artifact.

## Conclusions

Even though the epidemiology and characterization of strains inhabiting the anterior nares as well as the characterization of gene expression profiles under *in vitro* conditions mimicking those *in vivo* have established a sound knowledge base of *S. aureus* colonization strategies, the mechanisms used by *S. aureus in vivo* to survive in the nasal environment are still poorly understood^19^,^49^. The comparison between *in vivo* gene expression profiles across different human hosts and then comparing those with published *in vitro* expression profiles has shown that the host environment has a stronger impact on the expression profile than growth phase or medium composition, supporting the notion that understanding colonization by pathogenic or commensal microorganisms as well as of infection processes necessitates *in vivo* studies. The differences in gene expression profiles between samples has revealed diverse mechanisms used by *S. aureus* to persist in the human nose. As previously described^19^,^22^,^32^, survival in the nose is strongly controlled by the limitation of iron and evident by the expression of iron acquisition systems (*fep*ABC, *sir*ABC, *hts*ABC). Besides the need to acquire iron, *S. aureus* cells clearly experience different environmental stresses, which they commonly attempt to overcome by the expression of compatible solute biosynthetic pathways, changes in the cell wall composition and the synthesis of general stress proteins. Moreover, the expression of adhesins is important for colonization of the nares as evident by the consistent expression of *sdr*CDE*, clfB* and *ebpS in vivo*. However, different *S. aureus* strains also showed different *in vivo* behavior, maybe also due to differences in the environment set by the host. This is most evident for adhesins, where *sas*G expression was dominant in only one volunteer. Overall the analysis of general expression patterns and commonalities between different *S. aureus* strains in distinct host environments can provide new knowledge to base strategies to combat colonization by *S. aureus* or other opportunistic pathogens.

## Materials and Methods

### Confirmation and characterization of persistent *S. aureus* carriers

Persistent colonization of putative *S. aureus* carriers was analyzed by inoculation of cotton swabs of the anterior nares of volunteers on selective chromogenic media (CHROMagar^TM^ Staph aureus) and incubation for 48 h at 37 °C in ambient air. Suspicious colonies were confirmed as *S. aureus* by amplification of the *nuc* gene as previously described^50^. Isolated colonies from two nasal swabs from persistent carriers (those where *S. aureus* was observed at least four times in a one year period) were characterized by sequencing of the repeat region of the protein A gene (*spa*)^51^. The spa types were assigned through the Ridom web server (http://www.spaserver.ridom.de/). As from the different carriers always the same spa type was observed, further identification of a single isolate was performed by Multilocus Sequence Typing (MLST) as previously described^52^ and the sequence type assigned using the MLST database (http://saureus.mlst.net). Three male volunteers aged 30–50 years colonized in their anterior nares by *S. aureus* were thereby selected for the present study. Informed consent was obtained from all three volunteers included in this study for the analyses described. The study was approved by the Ethical Committee of the Medical Faculty of the University of Münster and of the Ärztekammer Westfalen-Lippe (file number 2010-468-f-S) and was carried out in accordance with the relevant guidelines.

### Total RNA extraction, purification and mRNA enrichment

Samples for metatranscriptomic analysis were taken at two time points (February and August 2013) using dry sterile cotton swabs. As only small amounts of RNA could be obtained from a single nasal swab, RNA from consecutive swabs was pooled. To verify the stability of the microbial community over short period of times, single swabs were taken daily from both anterior nares of a volunteer and the microbial community structure profiled by T-RFLP (terminal restriction fragment length polymorphism) as previously described^53^. After confirming the stability of the community over short time frames, swabs were taken from both anterior nares of each volunteers in the morning and evening of five consecutive days. Samples were stored at −80 °C after addition of 100μl of RNA-later^®^.

Single samples (swabs) were resuspended in 1 ml of cold buffer RLT (Qiagen) supplemented with 1% β-mercaptoethanol (Sigma) and transferred to lysing matrix B tubes (MP Biomedicals) on ice. Samples were disrupted using the FastPrep-24^®^ instrument (MP Biomedicals) at an intensity of 5.5 for 40 s. Samples were returned to ice for 4 min, disrupted further using the same settings and then centrifuged at 13 500 × *g* for 10 min at 4 °C to remove the cell debris. The supernatant was subsequently transferred to 1.5 ml RNase free Biopur™ centrifuge tubes (Eppendorf) and the RNA extracted using the RNeasy Mini Kit according to the manufacturer’s instructions, including optional DNase treatment on the column (Qiagen). RNA was eluted with nuclease-free water (Ambion), pooled and concentrated by ethanol precipitation using standard procedures.

### Amplicon library preparation and analyses

An aliquot of each pooled RNA sample was transformed into cDNA using the QuantiTect^®^ reverse Transcription kit (Qiagen) following the manufacturer instructions and 16S rRNA amplicon libraries comprising the V1-V2 variable regions were prepared as previously described^54^. These 6 samples were sequenced on the MiSeq instrument (Illumina) with processing of sequence reads performed as previously described^54^. In brief, quality filtered reads were trimmed conservatively to 80 nt and the paired ends matched to give 160 nt for downstream analysis. Reads were clustered allowing for two mismatches^55^ and the dataset then filtered to consider only those phylotypes that were present in at least one sample at a relative abundance >0.05% of the total sequences of that sample. The mean number of sequences per sample was 11345 +/− 2482 totaling 68072 sequence reads and the taxonomic affiliation of the 142 phylotypes was assigned as previously described^54^.

### Transcript library construction and sequencing

mRNA was enriched from each of the 6 samples using Terminator™ 5′-Phosphate-Dependent Exonuclease (Epicentre) according to the manufacturer’s instructions. Samples were further concentrated by ethanol precipitation and re-eluted in nuclease-free water (Ambion). RNA integrity and quantity was measured using the Agilent 2100 Bioanalyzer (Agilent Technologies) and NanoDrop 1000 spectrophotometer (Thermo Scientific). Libraries were generated using the ScriptSeq™ v2 RNA-Seq Library Preparation Kit (Epicentre). For each library, 50 ng of enriched mRNA was used in each reaction according to the manufacturer’s instructions and the libraries purified using the Minelute PCR Purification Kit (Qiagen). The libraries were further purified for removal of potentially contaminating primer dimers by agarose gel electrophoresis and excision and purification of the 250–650bp fragments by the QIAquick Gel extraction Kit (Qiagen). Libraries were assessed for quality using the Agilent 2100 Bioanalyzer (Agilent Technologies) and were sequenced on the Illumina HiSeq 2500 platform using the TruSeq SR Cluster Kit v3-cBot-HS (Illumina). Four libraries were multiplexed per lane (12 pM/library) and sequenced to 200 cycles in both directions.

### RNA-Seq data processing

Each library produced between 50 and 105 million reads, which were pre-processed for quality and trimmed using a combination of in-house Ruby scripts and open source tools (http://bioinformatics.ucdavis.edu/index.php/Trim.pl and http://www.mothur.org/)^56^. Reads were collapsed into representative reads using FASTX toolkit V. 0.0.13.2 collapser (http://hannonlab.cshl.edu/fastx_toolkit/).

Human associated RNAs were identified and removed from the *in vivo* sample using BLAT searches^57^ of an in-house database of the human genome repository (human RefSeq, chromosome records with gap adjusted concatenated NT_contigs) downloaded from the NCBI ftp site, blastdb (March 2013). Reads with alignment coverage (on the query) ≥30% were removed from the dataset. Ribosomal sequences contained within the datasets were detected with HMMER (version 3.0^58^) using models based on multiple sequence alignments of the 5S, 16S and 23S rRNAs^59^ and by the use of riboPicker (version 0.4.3). Results of the two methods were compiled and used to depurate datasets.

Seven nucleotide sequence databases were constructed and used to assign metatranscriptomic reads. These databases comprised the complete genomes from 1) the 25 *S. aureus* strains^24^, 2) *S. epidermidis* strains ATCC12228 and RP62A, 3) *F. magna* (ATCC29328), 4) *M. catarrhalis* RH4, 5) *P. acnes* SK137, 6) *D. pigrum* ATCC51524, 7) *Corynebacterium accolens* ATCC49725 and *Corynebacterium variabile* DSM44702 and 8) *Peptoniphilus duerdenii* (ATCCBAA164). The metatranscriptomic datasets devoid of human and ribsosomal reads were individually analyzed using this set of blast databases. Reads were assigned to a genus, if the ratio (alignment length * % identity)/ query length) exceeds 80%. As a reasonable number of reads could not clearly be assigned to either *S. aureus* or *S. epidermidis*, a ratio (alignment length * % identity)/ query length) >90% was used to assign reads to these species (see Table 1).

Presumptive *S. aureus* reads were mapped against a previously described *S. aureus* OG/Position Specific Scoring Matrix (PSSM) database^24^ using rpstblastn. An alignment ratio of ≥60% (alignment length * % identity/ query length) was used for the positive assignment of sequences to the orthologous groups. Reads that mapped to *S. aureus* were further blasted (blastn) against two genomes of *S. epidermidis (S. epidermidis* strains ATCC12228 and RP62A). Reads that showed an alignment ratio ≥90% were aggregated and assigned as reads that could originate from either S. aureus or S. epidermidis (Supplementary Dataset S2).

As the number of *S. aureus* sequence reads varied between libraries (see Table 2) and then to compare with recently published *in vitro* data that comprised orders of magnitude more sequence reads^24^, mapped read counts were re-randomized to the smallest library size of 5000 reads using an in-house Perl script^20^. Finally all read count data were normalized to reads per million (rpm).

Clusters of Orthologous Groups (COGs) were assigned to the probable *S. aureus* reads by querying the COG profiles deposited in the Conserve Domain Database (CDD) database^60^ (downloaded from the NCBI ftp site/ little endian) using rpsblast (version 2.2.25). The results were parsed using an in-house Perl script.

### Data analysis and interpretation

In order to compare the different *in vivo S. aureus* transcriptomic libraries with each other and then also with other published datasets (i.e. the *in vitro* libraries from Chaves-Moreno *et al*.^24^), a sample-similarity matrix was generated using the Bray–Curtis coefficient^61^ and gene-expression profiles compared using group-average agglomerative hierarchical clustering using PRIMER (v.6.1.6, PRIMER-E, Plymouth Marine Laboratory, Plymouth, UK)^62^.

Previous analysis of *S. aureus* 6850^20^ and *S. aureus* SH1000 global transcriptomic profiles using the ScriptSeq™ v2 RNA-Seq Library Preparation Kit (as performed here), showed similarities of 80–85% between replicates. Then, a direct comparison between the global transcriptomic profiles obtained from *S. aureus* SH1000 cells grown to mid-log phase in BHI using the MessageAmp™ II-Bacteria RNA amplification kit (Ambion) with a T7 primer modified to include a BpmI restriction site for removal of poly-A tails prior to sequencing as described by Chaves-Moreno *et al*.^24^ or the ScriptSeq™ v2 RNA-Seq Library Preparation Kit (Epicentre) (like the *in vivo* samples of this work) revealed global expression profiles of 75% similarity (using the Bray-Curtis algorithm) with a Spearman rank correlation of 0.911 (based on the read counts of the 2,582 *S. aureus* genes). So we can deduce that the choice of RNAseq library preparation method may contribute to an additional 10% difference in the global expression profile while retaining the rank-order of gene expression.

As the *in vivo* transcriptomes were very different and cannot be regarded as replicates, a detailed comparison with previous *in vitro* data^24^ was based on those genes that were expressed in at least one *in vivo* sample at a level exceeding 1000 rpm (Supplementary Fig. S1). Only genes where the expression level under at least one *in vitro* condition differed to that of the mean *in vitro* expression level of *S. aureus* USA300 strain LAC and *S. aureus* IPL32 growing in SNM or BHI, respectively, by at least one order of magnitude were considered for discussion in this paper (see Supplementary Dataset S3). Genes where the % of reads that also map to *S. epidermidis* exceeded 50% under any condition were not considered. Overall 240 genes were further compared.

### Data accessibility

Sequences generated from these 6 *in vivo* samples were deposited in the NCBI Gene Expression Omnibus (GEO) repository under the accession number GSE73485.

Nucleotide sequences of all 142 phylotypes determined using Illumina-based amplicon deep-sequencing, their relative abundance (in%) and their phylogenetic assignment, an overview of the expression of *S. aureus* genes under *in vivo* conditions analyzed here compared to previously described *in vitro* conditions and an overview of genes where the expression level under at least one *in vitro* condition differed to that previously described *in vitro* are provided as Supplementary Dataset S1,S2.

## Additional Information

**How to cite this article**: Chaves-Moreno, D. *et al*. Exploring the transcriptome of *Staphylococcus aureus* in its natural niche. *Sci. Rep.* **6**, 33174; doi: 10.1038/srep33174 (2016).

## Supplementary Material

Supplementary Information

Supplementary Dataset S1

Supplementary Dataset S2

Supplementary Dataset S3

## Figures and Tables

**Table 1:** Bioinformatic processing of the sequence reads generated from the 6 metatranscriptomic libraries.

Bioinformatic filtering step	1W	1S	2W	2S	3W	3S
raw reads	46,299,650	47,487,990	9,921,650	41,258,105	59,204,687	114,236,035
reads after quality filtering	42,037,251	44,693,474	8,972,017	34,585,687	53,143,044	106,548,415
reads after removal of human reads	3,440,056	5,822,346	4,175,206	29,070,525	2,620,088	4,109,102
mRNA reads after removal of ribosomal reads	2,967,566	3,524,160	2,441,919	8,366,998	1,919,599	2,850,659
reads assigned exclusively to *S. aureus*, similarity^1^ ≥80%	5,603	3,544	33,858	5,1363	10,372	33,869
reads assigned exclusively to *S. epidermidis*, similarity ≥80%	9,169	33,339	17,048	86,094	1,075	5,259
reads assigned to both, similarity ≥80%	6,411	28,978	21,304	114,967	5,528	18,164
reads assigned exclusively to *S. aureus,* similarity ≥90%	6,342	817	39,512	70,016	13,036	43,310
reads assigned exclusively to *S. epidermidis,* similarity ≥90%	11,456	47,930	22,475	133,478	1,472	7,077
reads assigned to both, similarity ≥90%	2,040	7,806	5,901	37,247	2,044	4,776
reads mapped exclusively to genes of *S. aureus,* similarity ≥90%	4,853	583	30,592	55,517	10,165	34,984
reads mapped exclusively to genes of *S. epidermidis,* similarity ≥90%	9,137	38,554	18,009	111,611	897	5,664
reads mapped to both, similarity ≥90%	944	5,464	3,861	26,733	1,226	3,464
% of reads assigned to *S. aureus* (similarity ≥90%) that could be mapped	73	70	77	79	76	80

^*^Similarity of alignment length * % identity/ query length ≥80% or ≥90%.

**Table 2:** Assignment of mRNA reads to the key bacterial species (or genera) within the human anterior nares.

Reads assigned to	1W	1S	2W	2S	3W	3S
mRNA	2,967,566	3,524,160	2,441,919	8,366,998	1,919,599	2,850,659
*S. aureus,* similarity* ≥90%	6,342	817	39,512	70,016	13,036	43,310
*S. epidermidis,* similarity ≥90%	11,456	47,930	22,475	133,478	1,472	7,077
*Corynebacterium spp,* similarity ≥80%	16,855	153,932	35,386	73,409	1,589	5,024
*D. pigrum,* similarity ≥80%	114	139,266	477	2,890	92	637
*F. magna,* similarity ≥80%	2,334	6,985	12,719	18,570	272	1,429
*Peptoniphilus spp,* similarity ≥80%	1,083	3,875	5,275	10,505	86	557
*P. acnes,* similarity ≥80%	8,621	21,772	63,422	104,072	1,368	12,953
total of reads assigned to above bacteria	45,582	36,7543	176,840	309,796	18,158	70,886
% of potential mRNA reads that were assigned to above bacteria	1.7	11.2	7.8	5.8	1.1	2.8

^*^Similarity of alignment length * % identity/ query length ≥ 80% or ≥90%.
