# Supplementary material for: Exploring the transcriptome of Staphylococcus aureus in its natural niche
Source: Sci Rep. 2016 Sep 19;6:33174. doi: 10.1038/srep33174 (PMC5027550; doi:10.1038/srep33174)
Supplement: Supplementary Information [file srep33174-s1.pdf]

Exploring the transcriptome of *Staphylococcus aureus* in its natural niche

Diego Chaves-Moreno[1], Melissa L Wos-Oxley [1], Ruy Jáuregui[1,3], Eva Medina[2], Andrew PA Oxley[1,4], Dietmar H Pieper[1*]

[1]Microbial Interactions and Processes Research Group, Helmholtz Centre for Infection Research, Inhoffenstr. 7, 38124 Braunschweig, Germany

[2]Infection and Immunology Research Group, Helmholtz Centre for Infection Research, Inhoffenstr. 7, 38124 Braunschweig, Germany

[*] For correspondence: Dietmar H. Pieper, Microbial Interactions and Processes Research Group, Helmholtz Centre for Infection Research, Inhoffenstr. 7, 38124 Braunschweig, Germany. E-mail dpi@helmholtz-hzi.de; Tel. +49 531 6181 4200

[3] Present address: AgResearch, Tennent Drive, Palmerston North 4442, New Zealand

[4]Present address: Molecular Sciences Laboratory, SARDI Aquatic Sciences, 2 Hamra Ave, West Beach, South Australia, Australia

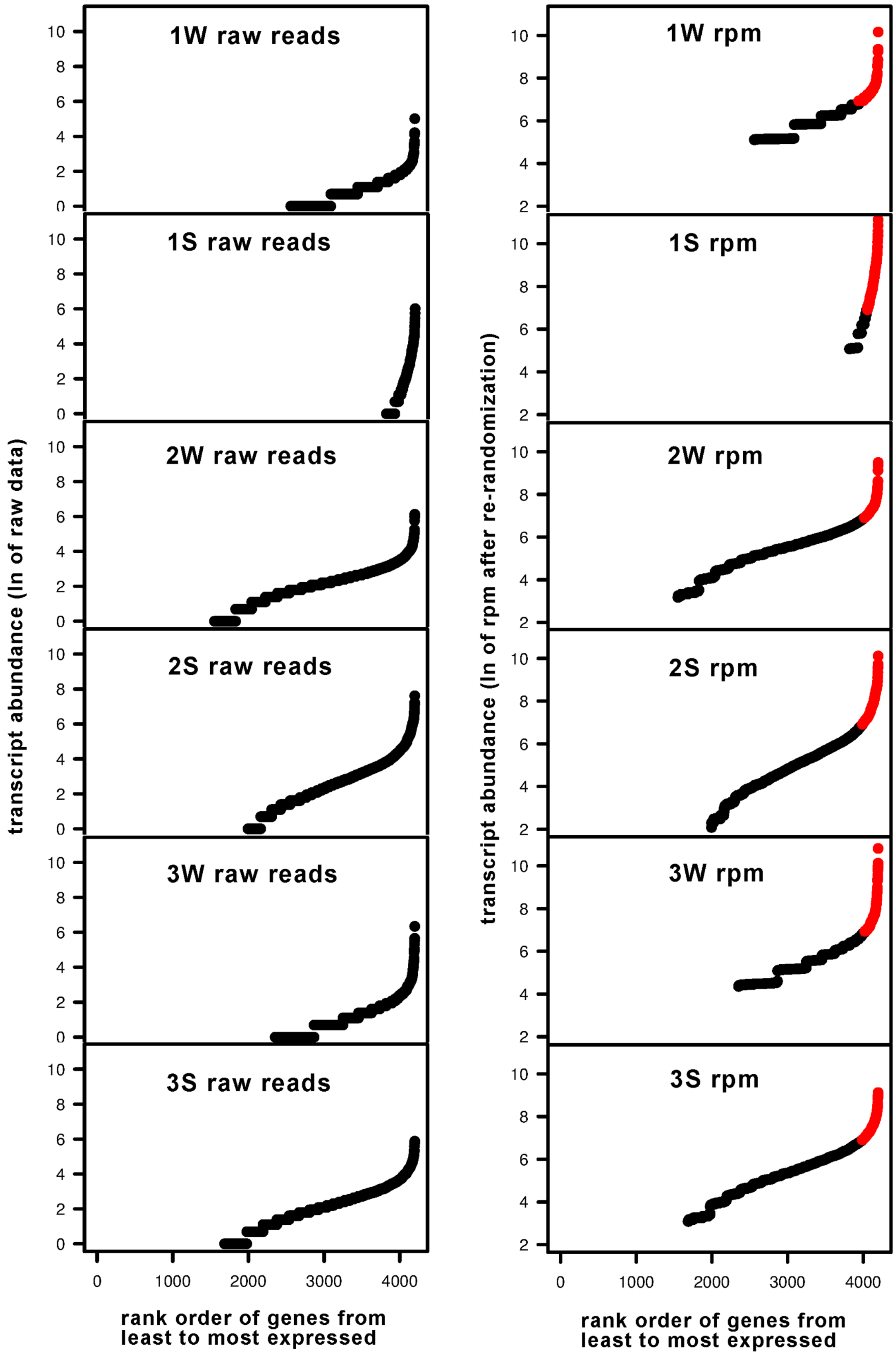

Fig. S1. Rank-ordered distributions of *S. aureus* gene expression levels under *in vivo* conditions. Plots of raw read data (left) and reads per million (rpm, after re-randomization, right). Genes expressed at levels >1000 rpm are shown in red.

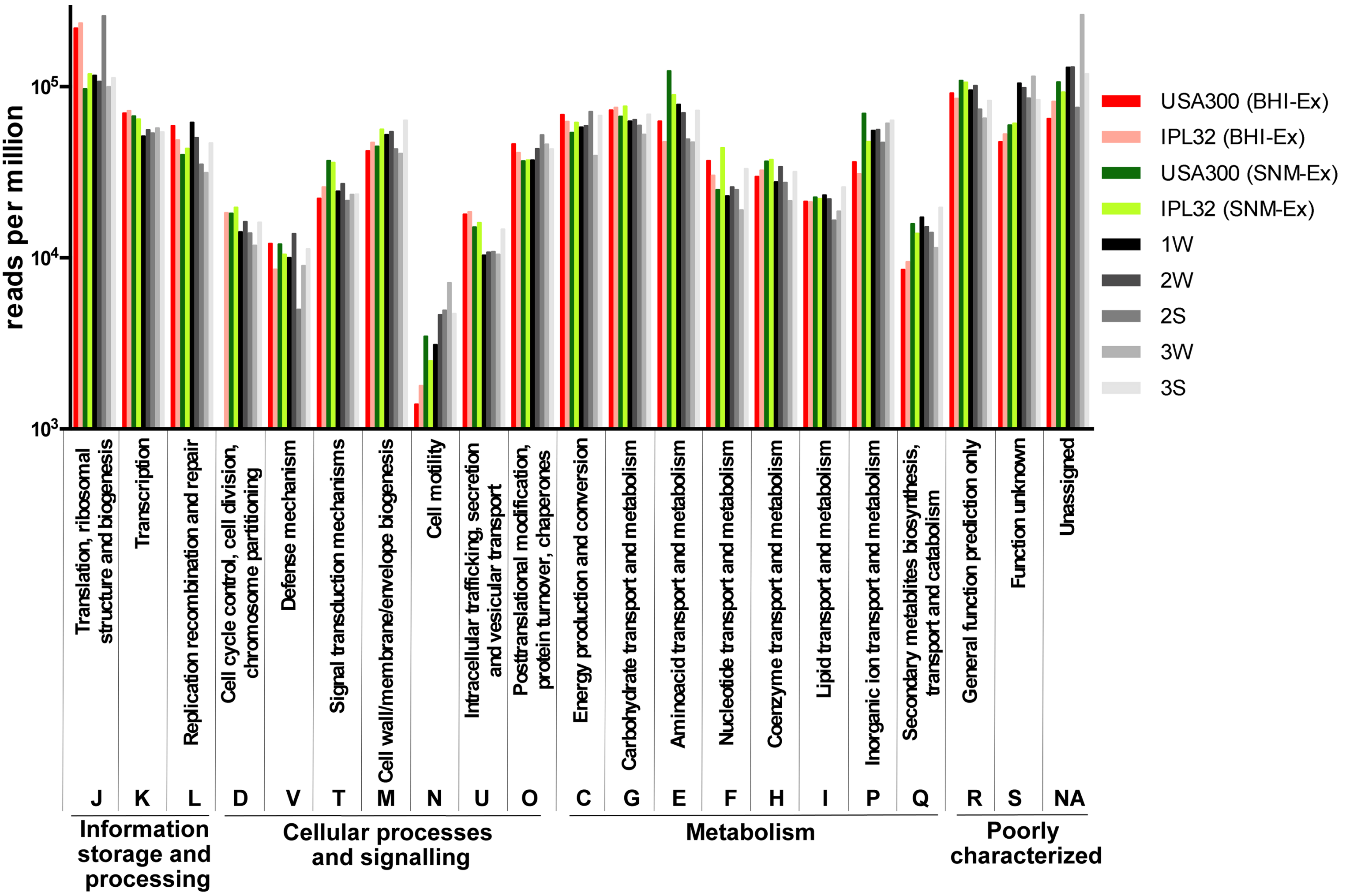

Fig. S2. Distribution of the abundances of transcripts assigned to the major Clusters of Orthologous Groups categories (X axis) during exponential growth of *S. aureus* USA300 LAC and IPL32 i*n vitro* in Brain Heart Infusion (BHI) or Syntheic Nasal Medium (SNM) and i*n vivo* in three different volunteers. Number of reads assigned per category are given as reads per million.
